# Effect of Post-Heat Treatment on Microstructure and Corrosion Property of Additively Manufactured AlCoCrFeNi_2.1_ Eutectic High-Entropy Alloy

**DOI:** 10.3390/ma18245544

**Published:** 2025-12-10

**Authors:** Xinping Li, Hao Ding, Xinyue Pi, Shuying Zhang, Yun Xie

**Affiliations:** 1 School of Engineering Technology, Nanchang Vocational University, Nanchang 330500, China; 2 School of Materials Science and Engineering, Nanchang Hangkong University, Nanchang 330063, China

**Keywords:** additive manufacturing, eutectic high-entropy alloy, heat treatment, microstructure, corrosion resistance

## Abstract

In the present study, AlCoCrFeNi_2.1_ eutectic high-entropy alloy (EHEA) was fabricated by laser melting deposition (LMD). Then, post-heat treatment was performed at different temperatures to investigate its effects on microstructure and corrosion property of the alloy. The results obtained from microstructural characterization indicate that the alloy, whether heat-treated or not, exhibited a lamellar eutectic microstructure composed of alternating FCC and BCC phases. With the increase in the heating temperature from 600 to 1000 °C, the interlamellar spacing and volume fraction of the FCC phase gradually increased. Electrochemical testing in 3.5 wt.% NaCl solution revealed that the resistance of the alloy to corrosion was improved with the increasing heating temperature, which was attributed to the increased volume fraction of the FCC phase. However, the immersion test in 3.5 wt.% NaCl solution also suggests that heating above 800 °C increased the susceptibility of the alloy to pitting corrosion, due to the more pronounced enrichment of Al in the BCC phase.

## 1. Introduction

Since their advent in 2004 [1,2], high-entropy alloys (HEAs) have attracted considerable attention in the materials research field, owing to their distinctive compositional characteristics, microstructural features, and tunable properties [3,4]. To address the strength–ductility trade-off commonly found in conventional single-phase face-centered cubic (FCC) or body-centered cubic (BCC) HEAs, Lu et al. [5] initially developed AlCoCrFeNi_2.1_ eutectic high-entropy alloy (EHEA), which featured a dual-phase lamellar eutectic microstructure comprising hard BCC and soft FCC phases. Accordingly, this alloy possesses a good combination of strength and ductility, and thereby it is viewed as a promising candidate material for a wide range of engineering applications [6,7,8].

In recent years, preparing AlCoCrFeNi_2.1_ EHEA by laser additive manufacturing (LAM) has become popular, as this revolutionary technology allows for the lightweight, personalized, and integrated manufacturing of geometrically complex components [3,9,10]. However, the extremely rapid heating and cooling rates inherent in the LAM process can generate significant temperature gradients, resulting in typical defects such as porosity, inclusion, and lack-of-fusion defects [11,12]. These defects are likely to serve as initiation sites for localized pitting corrosion, intergranular corrosion, and stress corrosion cracking, which is particularly pronounced in aggressive environments [13]. Unfortunately, currently available research concerning the LAM-ed AlCoCrFeNi_2.1_ EHEA principally concentrates on its microstructure and mechanical properties [3,7,14,15,16], while the studies on its corrosion characteristics are limited, in particular for the influence of post-heat treatment on its corrosion performance. Zhou et al. [17] reported that due to the thermal cycling and rapid solidification associated with selective laser melting (SLM), the homogeneous elemental distribution between the FCC and BCC phases in SLM-ed AlCoCrFeNi_2.1_ EHEA was conducive to the corrosion property. Luo et al. [18] discovered increasing volumetric energy density reduced the volume fraction of the BCC phase in the SLM-ed AlCoCrFeNi_2.1_ EHEA, resulting in the enhanced corrosion resistance.

In this study, AlCoCrFeNi_2.1_ EHEA was prepared by laser melting deposition (LMD) with optimized laser energy density on a basis of our preliminary research findings [19]. Electrochemical methods, XRD, and SEM/EDS were then employed to investigate the microstructure and corrosion property of AlCoCrFeNi_2.1_ EHEA in 3.5% NaCl solution after heat treatment at different temperatures. The specific components of the corrosion product were analyzed using XPS. The findings of this work are anticipated to benefit the design of post-heat treatment of LAM-ed AlCoCrFeNi_2.1_ EHEA and meanwhile shed some light on the elucidation of processing–microstructure–performance relationship of this alloy.

## 2. Materials and Methods

**Figure 1:**
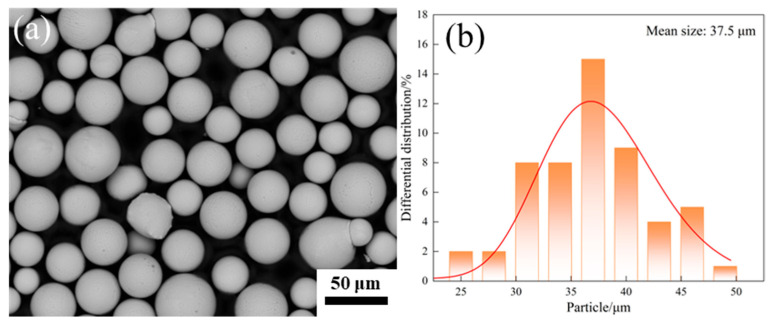
(**a**) Morphology of AlCoCrFeNi_2.1_ powders, (**b**) particle size distribution.

The AlCoCrFeNi_2.1_ alloy powders prepared by plasma rotating electrode process (PREP) were provided by Beijing Yanbang New Material Technology Co., Ltd. (Beijing, China). As shown in Figure 1, the powders exhibit good sphericity, with the size located in the range of 15~53 μm. The powder chemical composition determined by energy-dispersive X-ray spectroscopy (EDS) is provided in Table 1, indicating a good agreement with the atom ratio Al:Co:Cr:Fe:Ni = 1:1:1:1:2.1. The powders were subjected to drying at 100 °C for a minimum of 4 h to eliminate moisture prior to their use.

The AlCoCrFeNi_2.1_ EHEA specimens with the size of 10 mm × 10 mm × 5 mm were fabricated on a carbon steel substrate using a CO_2_ laser system. The main processing parameters were as follows: the laser power was 800 W, the scanning speed was 1200 mm/min, the spot diameter was 2.2 mm, the powder feeding rate was 2.3 g/min, the track spacing was 0.5 mm, and the protective gas flow rate was 20 L/min. Both the protective gas and the powder supply gas were high-purity Ar gas (99.9%). A meander laser scan path pattern was adopted with a hatch spacing of 40%. To minimize the internal residual stress, the path frame of reference was rotated by 36° with each layer added.

The AlCoCrFeNi_2.1_ EHEA specimens having a thickness of about 2~3 mm were cut from the substrate by wire electrical discharge machining. As shown in Table 2, post-heat treatment was performed by heating the specimens to 600 °C (2#), 800 °C (3#), and 1000 °C (4#), holding in 95%Ar + 5%H_2_ for 4 h, followed by cooling in the furnace. The as-prepared specimen was marked as 1#. Subsequently, the specimen surfaces were ground successively with SiC paper up to 2000 mesh for ongoing tests.

Microstructural and compositional characterization was performed with X-ray diffraction (XRD) with Cu-Kα radiation and a scanning electron microscope (SEM) equipped with energy-dispersive X-ray spectroscopy (EDS).

Electrochemical tests were conducted on an electrochemical workstation (CS310H, Corrtest Instruments, Wuhan, China) using a standard three-electrode system in 3.5 wt.% NaCl solution. In this system, a saturated calomel electrode (SCE), a platinum sheet, and an AlCoCrFeNi_2.1_ EHEA sample with an exposed area of 1 cm^2^ served as the reference electrode, the counter electrode, and the working electrode, respectively. The exposed surface was polished down to a 0.5 μm finish and ultrasonically cleaned in ethanol. Prior to the electrochemical measurements, a constant potential polarization at −0.8 V for 60 s was applied on the working electrode to eliminate the superficial oxide layer. Next, the open circuit potential (OCP) was monitored for 1 h until a stable state was reached. Subsequently, the electrochemical impedance spectroscopy (EIS) test was performed in the frequency range of 100 kHz to 10 mHz, and the AC amplitude was ±5 mV. The EIS data were fitted and analyzed using ZsimpWin 2.70 software. The scanning rate and initial potential of potentiodynamic polarization test was kept at 1 mV/s and −0.5~0.5 V (vs. OCP).

The corrosion morphology of the specimens immersed in 3.5 wt.% NaCl solution at room temperature for 48 h was examined with the above-mentioned SEM/EDS and X-ray photoelectron spectrometer (XPS). The specimens were cleaned by distilled water and alcohol before examination.

## 3. Results and Discussions

### 3.1. Phase Composition and Microstructure

**Figure 2:**
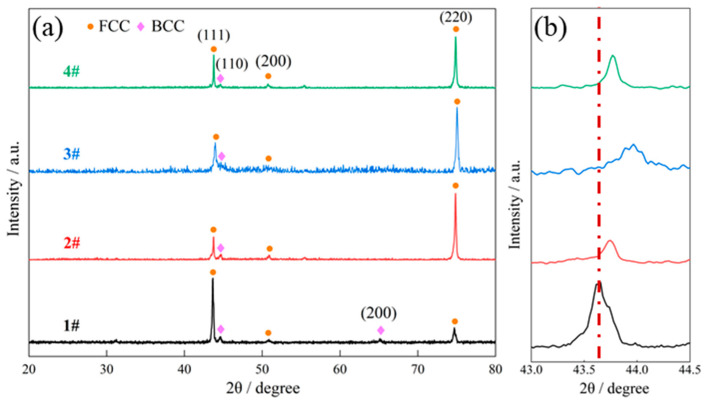
(**a**) XRD patterns of the four samples, (**b**) local enlargement of (**a**).

Figure 2 displays the XRD patterns of the as-prepared and heat-treated samples. Obviously, the AlCoCrFeNi_2.1_ EHEA, regardless of its state, was composed of FCC + BCC two phases, congruent to the previous research elsewhere [16,20,21]. In addition, there was no phase change in the four samples, suggesting the good phase stability of the AlCoCrFeNi_2.1_ EHEA at high temperatures. As can be seen, the intensity of the FCC phase peaks was consistently greater than that of the BCC phase, which indicates the dominance of the FCC phase over the BCC phase in the four samples.

As indicated by the locally enlarged view of the XRD patterns in Figure 2b, the position of the FCC peak located close to 2θ ≈ 44° slightly shifted towards a high angle as the heating temperature increased to 800 °C, indicating the decrease in atomic spacing. By contrast, it has been frequently reported [22,23,24] that the rapid cooling rate during the LAM process usually results in severe lattice distortion which expands atomic spacing in as-prepared alloys. Therefore, it can be deduced that increasing the temperature of post-heat treatment can more effectively alleviate the lattice distortion generated during the rapid solidification of the melt pool. Nevertheless, it is noted that as the heating temperature was further elevated to 1000 °C, the aforementioned FCC peak somewhat shifted slightly back to a low angle, suggesting the increase in the lattice constant of the FCC phase. The specific reason for this change is unknown in the present study.

**Figure 3:**
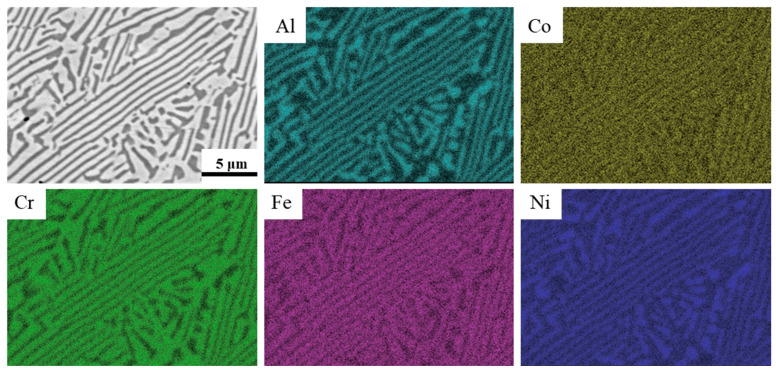
BSE-SEM image and elemental distribution of the as-prepared AlCoCrFeNi_2.1_ EHEA.

Figure 3 shows the backscattered electron (BSE)-SEM image and corresponding elemental distribution of the as-prepared AlCoCrFeNi_2.1_ EHEA. It is clear that the alloy exhibited a typical eutectic configuration, characterized with a lamellar biphasic microstructure comprising the alternating growth of a wide bright contrast FCC phase and narrow dark contrast BCC phase. In addition, the EDS mapping result suggests that the BCC phase was rich in Al and Ni, while the FCC phase was rich in Cr and Fe. As for Co, its enrichment in the FCC phase relative to in the BCC phase was very slight, exhibiting an almost uniform distribution through the two phases. These microstructural and compositional characteristics agree well with the previous reports [3,8,16].

**Figure 4:**
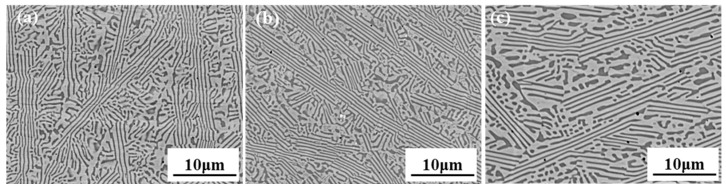
BSE-SEM images of the specimens: (**a**) 2#-600 °C-treated, (**b**) 3#-800 °C-treated, and (**c**) 4#-1000 °C-treated.

Figure 4 shows the BSE-SEM images of AlCoCrFeNi_2.1_ EHEA after heat treatment at different temperatures, with the examined surface perpendicular to the building direction. Apparently, the above-mentioned eutectic dual-phase microstructure of FCC and BCC in the as-prepared sample was retained for the three heat-treated samples. However, with the increase in the heating temperature, it is noted that the eutectic lamellae microstructure gradually coarsened and evolved into a spheroid or short-rod shape, especially for the 4# sample (Figure 4c).

**Figure 5:**
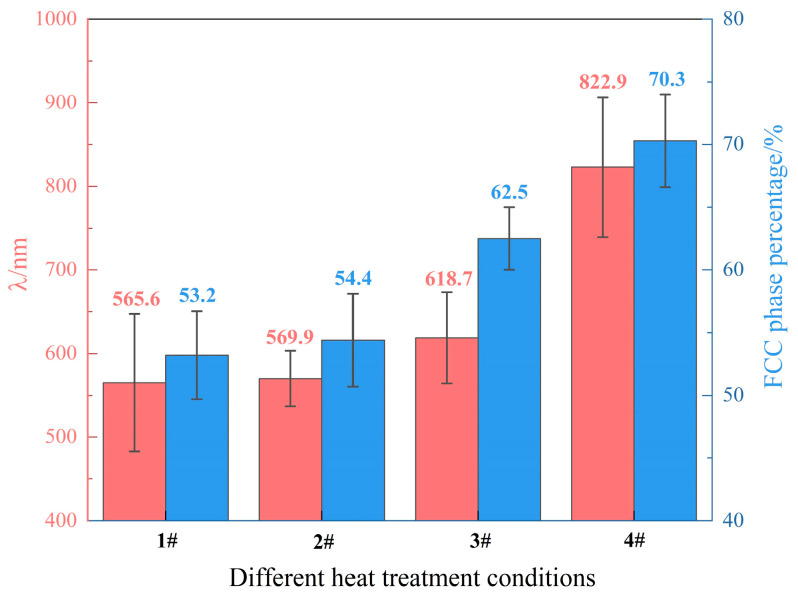
Interlamellar spacing (λ) and FCC volume fraction of the four samples.

To analyze the variation of the eutectic lamellae microstructure, the interlamellar spacing (λ) and volume fraction of the FCC phase in the four samples were measured by ImageJ 1.54p software and are displayed in Figure 5. The interlamellar spacing between adjacent FCC or BCC lamellae was measured following a previously established procedure [25], with an average value obtained from five randomly selected regions. Similarly, the FCC volume fraction in the four samples was determined by averaging measurements taken at five distinct locations. It shows that with the introduction of post-heat treatment and the continued increase in corresponding temperature to 1000 °C, the mean value of interlamellar spacing increased steadily from 565.6 to 822.9 nm. Meanwhile, the percentage of the FCC phase progressively increased from 53.2% to 70.3%. The consistently greater volume fraction of FCC compared with BCC in the four samples, whether heat-treated or not, is in accordance with the corresponding XRD patterns. It was reported [26] that the average volume fractions of FCC and BCC phases in as-cast AlCoCrFeNi_2.1_ EHEA were about 65% and 35%, respectively. Therefore, it can be deduced that applying post-heat treatment and elevating the corresponding temperature to 800 °C make the phase constitutions of the alloy closest to the equilibrium state, which is consistent with the result suggested by Figure 2b.

To further investigate the evolution of chemical composition of the FCC and BCC phases with the temperature of heat treatment, EDS analysis was conducted, with the results summarized in Table 3. It is worth noting that, with the elevation of the heating temperature, the Al content of the BCC phase generally increased, while the corresponding Cr content decreased. Nonetheless, a reversed trend was found for the Al and Cr contents in the FCC phase. For the 4# sample, which was heat-treated at 1000 °C, its BCC phase had a much higher Al content than the other three samples, whereas its FCC phase had the highest content of Cr. Since Al is a strong BCC phase stabilizer [27], heating at higher temperatures contributed to its enrichment in the BCC phase.

### 3.2. Electrochemical Properties

**Figure 6:**
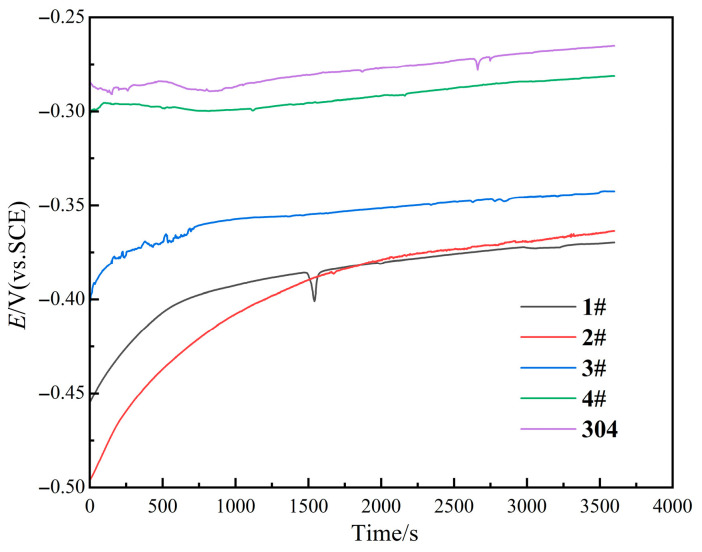
Open-circuit potential curves of the four samples and 304 stainless steel in 3.5% wt.% NaCl solution.

Figure 6 presents the evolution of OCP values of the four studied samples with the immersion time in 3.5% wt.% NaCl solution. In addition, the OCP curve of 304 stainless steel is also displayed in Figure 6 for comparison. It can be seen that each sample shows a gradually stable trend after 30 min.

**Figure 7:**
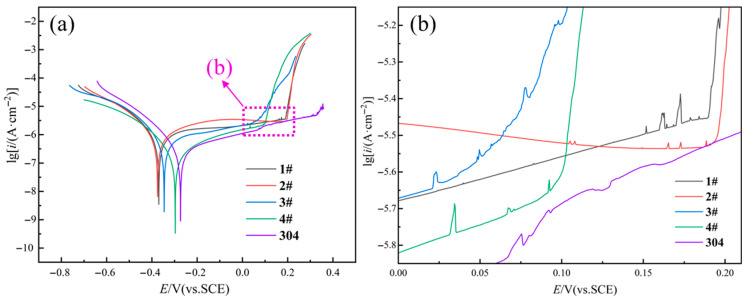
(**a**) Potentiodynamic polarization curves of the four samples and 304 stainless steel in 3.5 wt.% NaCl solution, (**b**) local enlargement of (**a**).

Figure 7a presents the potentiodynamic polarization curves of the four samples and 304 stainless steel in 3.5 wt.% NaCl solution, with the related values of corrosion potential (*E*_corr_) and corrosion current density (*i*_corr_) included in Table 4. Evidently, passivation was revealed by all the five curves in the anodic polarization region. Furthermore, no activation–passivation transition zone was observed in any polarization curves, indicating that a passive film was spontaneously developed by all the samples [28,29,30]. It is generally accepted that higher *E*_corr_ means, in terms of thermodynamics, a greater stability of the alloy in the corrosive medium, while lower *i*_corr_ suggests, from the view of kinetics, a smaller uniform corrosion rate. As revealed by Figure 7a and Table 4, with the increase in the heating temperature, *E*_corr_ slightly increased, while *i*_corr_ decreased, suggesting the improvement of corrosion resistance. This can be attributed to the increased volume fraction of the FCC phase (Figure 5), as previous studies [31,32] have reported that the BCC phase is preferential for corrosion due to the micro-galvanic couplings of the FCC and BCC phase. Specifically, the 4# sample showed the highest *E*_corr_ (−297 mV) and the lowest *i*_corr_ (3 × 10^−7^ A/cm^2^) among the four samples, which corresponded well with the smallest proportion (70.3%) of the FCC phase in this sample. The beneficial effect of the increased volume fraction of the FCC phase on the corrosion resistance was in good agreement with the findings reported by Luo et al. [18].

However, Figure 7b shows that 3# and 4# samples exhibited apparently lower pitting potential (*E*_pit_) than the other two samples, which indicates that the rupture of the passive film or the initiation of pitting corrosion was more prone to occur as the heat treatment temperature reached 800 °C and above. Furthermore, a small current peak was observed within the passive region of the 4# sample when the loading potential was still about 70 mV lower than its *E*_pit_, suggesting the emergence of metastable pits in the passive film [33]. This also suggests the increased susceptibility of the passive film developed by the 4# sample to pitting corrosion.

**Figure 8:**
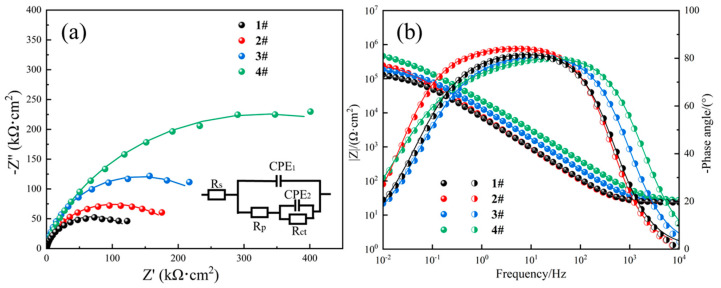
(**a**) Nyquist and (**b**) Bode plots of the four samples in 3.5 wt.% NaCl solution.

Figure 8 displays the Nyquist and Bode plots obtained by EIS testing of the four samples immersed in 3.5 wt.% NaCl solution at room temperature. As shown in Figure 8a, all the Nyquist curves displayed a single capacitance semi-arc, and the radius of capacitance arc of the 4# sample was maximal. Since the size of the semi-arc represents the corrosion resistance of materials in solution [30], it can be derived that the 4# sample shows the best corrosion resistance among the four studied samples, which is consistent with the results of the polarization curves. The Bode curves in Figure 8b show that the value of impedance modulus $\left|Z\right|$ tends to be constant at roughly 30 Ω·cm^2^ in the high-frequency range (10^3^~10^4^ Hz), and the phase angle approaches 0° with the increasing frequency, which reflects the resistive response of the solution resistance [34]. In the middle-frequency range, a linear relationship was displayed between the values of $\left|Z\right|$ and frequency, and the maximum phase angle was generally stabilized at about 80°, which manifests the passivation of the oxide film formed on the sample surface. In the low-frequency domain, the $\left|Z\right|$ value of the 4# sample was largest among the four samples, suggesting the best corrosion resistance [35].

The equivalent electrical circuit (EEC) model inserted in Figure 8a was used to fit the EIS data. In the EEC model, *R*_s_, *R*_f_, and *R*_ct_ represent the solution resistance, the resistance of the passive film, and the charge transfer resistance, respectively. CPE_1_ and CPE_2_ reflects the electrochemical response of the passive film and the electric double layer, respectively. The CPE (*Z*_CPE_) values can be calculated as follows:

(1) $$Z_{\mathrm{CPE}}=\frac{1}{Y_{0}{\left(\omega ,j\right)}^{n}}$$

where *Y*_0_ is the admittance value, *n* is fitted exponential which varies from 0 to 1, *ω* is the angular frequency, and *j* is the imaginary number. The relative fitting data are summarized in Table 5. The higher the polarization resistance, which equals the sum of *R*_f_ and *R*_ct_, the better the corrosion resistance [30]. According to the tabulated data, the sum of *R*_f_ and *R*_ct_ for the 4# sample is significantly larger than that for the other three samples, which also indicates that the 4# sample has the best corrosion resistance.

### 3.3. Corrosion Morphologies

**Figure 9:**
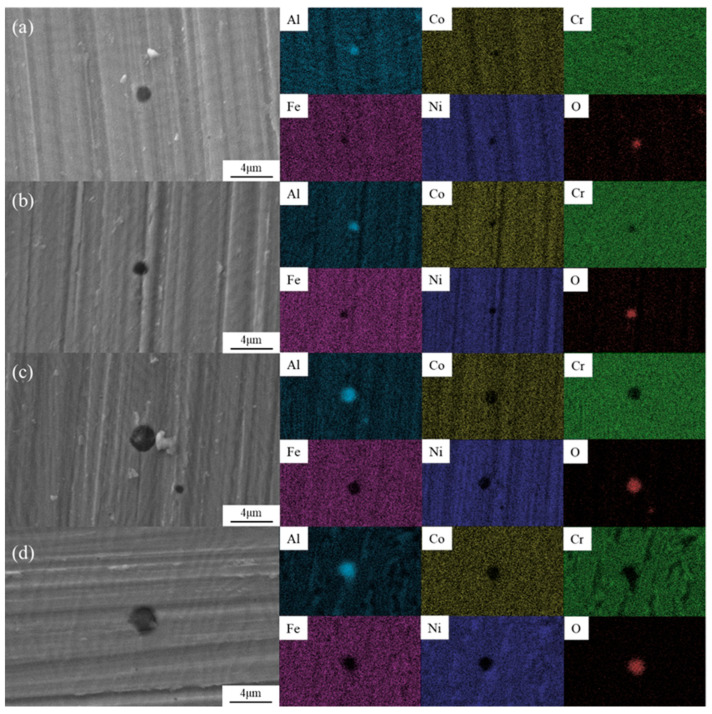
SEM/EDS images of the four samples after 48 h immersion in 3.5 wt.% NaCl solution: (**a**) 1#-Unheated treatment, (**b**) 2#-600 °C-treated, (**c**) 3#-800 °C-treated, and (**d**) 4#-1000 °C-treated.

Figure 9 displays the surface SEM morphology and EDS mapping results of the four samples after immersion in 3.5 wt.% NaCl solution for 48 h. Clearly, the four samples suffered pitting corrosion, and the size of corrosion pits in 1# and 2# samples (Figure 9a,b) was apparently smaller than that in 3# and 4# samples (Figure 9c,d). This result agrees well with the variation of *E*_pit_ suggested by Figure 7b and Table 4. In addition, as revealed by the EDS mapping results, all the corrosion pits were featured with the enrichment of Al-rich oxide, which could be identified as Al_2_O_3_ according to the research conducted by Song et al. [28].

Several studies have reported [28,31,36] that the Al-rich BCC phase is more prone to pitting corrosion, as Al_2_O_3_-rich film is relatively loose and more easily attacked by chloride ions (Cl^−^). On the contrary, Cr_2_O_3_ is more stable and compact than Al_2_O_3_, and thus the Cr-rich FCC phase remains almost unaffected during the immersion test. As shown in Table 3, the Al content in the BCC phase of 3# and 4# samples was higher than that in 1# and 2# samples, while a reversed trend was found for the corresponding Cr content. Therefore, it is explicable that Al_2_O_3_ content in the passive film grown on 3# and 4# samples is higher than that grown on 1# and 2# samples, and thus the former two are more easily damaged by Cl^−^. As a result, the varying pitting corrosion resistance of the different samples can be well related to their compositional difference.

It should be mentioned that the aforementioned elemental partition in the FCC and BCC phases was still somewhat marked for the 4# sample but not for the other three samples, which suggests that the corrosion film developed by the 4# sample was much thinner than that of the three counterparts. Therefore, this finding can be used to verify that the 4# sample exhibited the best resistance to uniform corrosion, which also has good consistency with the results of the polarization curves.

**Figure 10:**
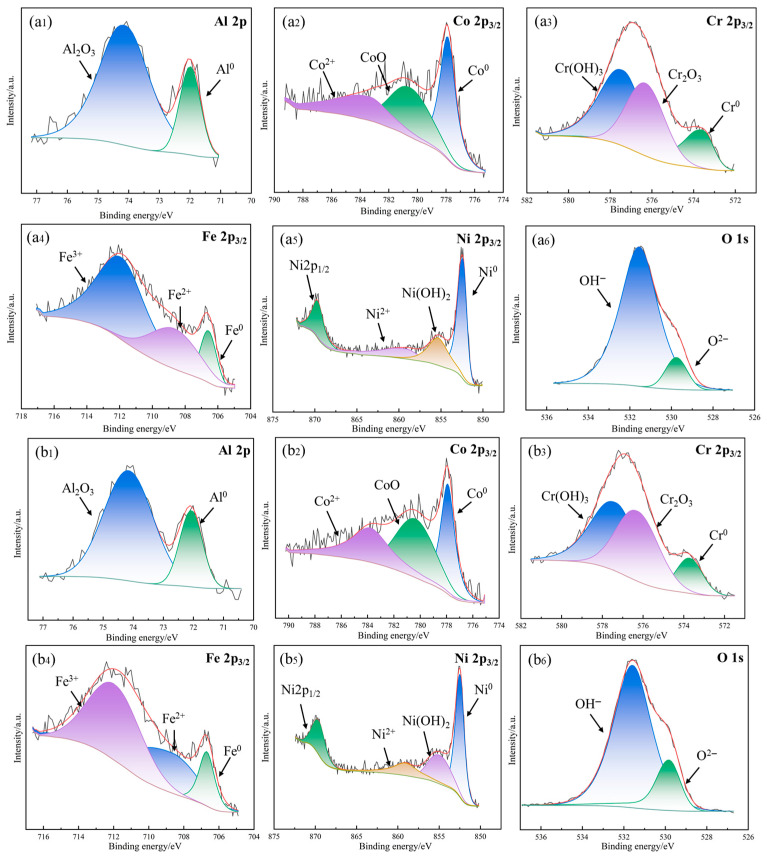

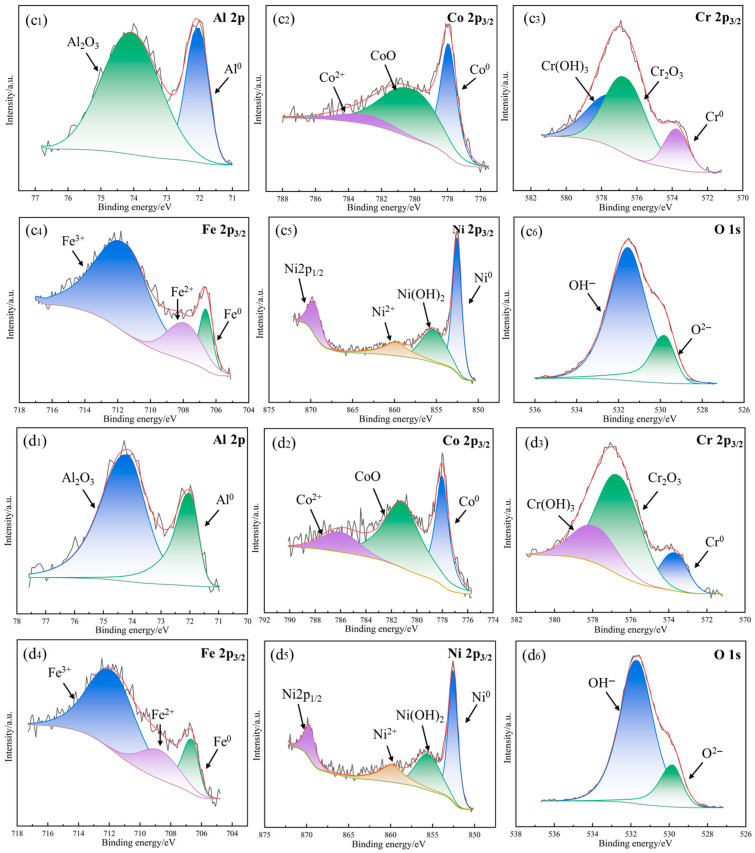
XPS spectra of the passive films recorded on the four samples after immersion in 3.5 wt.% NaCl solution for 48 h: (**a1**–**a6**) 1#-Unheated treatment, (**b1**–**b6**) 2#-600 °C-treated, (**c1**–**c6**) 3#-800 °C-treated, and (**d1**–**d6**) 4#-1000 °C-treated.

Figure 10 displays the XPS results of the four corroded samples after immersion in 3.5 wt.% NaCl solution for 48 h. It is apparent that the passivation films all include Al 2p, Co 2p_3/2_, Cr 2p_3/2_, Fe 2p_3/2_, Ni 2p_3/2_, and O 1s. Figure 10(a1,b1,c1,d1) show the Al 2p spectra collected from the passive films grown on the four specimens, and clearly the Al 2p spectrum is split into minor metallic Al and major Al_2_O_3_ peaks. Figure 10(a2,b2,c2,d2) show the XPS data of Co 2p_3/2_. The Co 2p_3/2_ peak data are divided into three peaks, which are Co^2+^, CoO, and Co. As shown in Figure 10(a3,b3,c3,d3), Cr 2p_3/2_ is decomposed into three peaks corresponding to metallic Cr, Cr_2_O_3_, and Cr(OH)_3_. It is worth noting that with the increase in heating temperature, the peak strength of Cr_2_O_3_ increases gradually, which indicates that elevating temperature increases the Cr_2_O_3_ content in the passivation film. As the dense Cr_2_O_3_ passive film can remain stable in 3.5 wt.% NaCl solution, the 4# sample exhibits the best corrosion resistance, which is consistent with the results shown in Figure 7. Figure 10(a4,b4,c4,d4) show the XPS data of Fe 2p_3/2_, which are divided into three peaks: Fe^3+^, Fe^2+^ oxide, and metallic Fe, and the intensity of the three peaks has no significant change. Figure 10(a5,b5,c5,d5) show the peaks of Ni 2p_3/2_, including Ni 2p_1/2_, Ni^2+^, Ni(OH)_2_, and metallic Ni. Metallic Ni is the main form of Ni 2p_3/2_, and the corresponding intensity of the four peaks almost remains unchanged. The O 1s spectrum is split into three peaks corresponding to O^2−^ and OH^−^, suggesting that the passivation film produced by AlCoCrFeNi_2.1_ mainly contains oxides and hydroxides.

**Figure 11:**
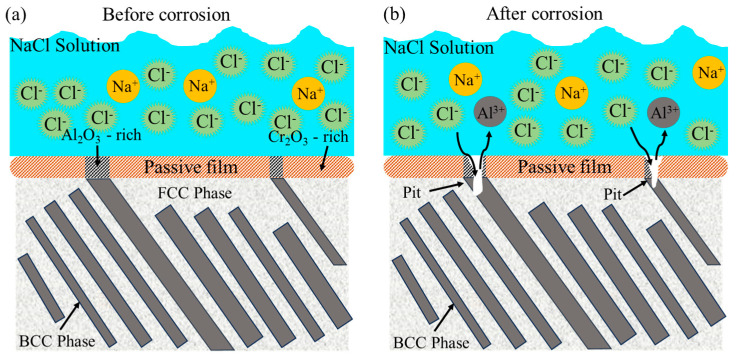
Schematic diagrams of the corrosion process of AlCoCrFeNi_2.1_ EHEA in 3.5 wt.% NaCl solution: (**a**) before corrosion, (**b**) after corrosion.

Figure 11 schematically illustrates the corrosion mechanism of AlCoCrFeNi_2.1_ EHEA in 3.5 wt% NaCl solution. Generally, a continuous passivation film is formed on the alloy surface (Figure 11a). However, due to the higher concentration of Al in the BCC phase than in the FCC phase, Al_2_O_3_ is locally enriched in the passivation film grown on the BCC phase, making it more vulnerable to the damage of Cl^−^. On the contrary, the passivation film formed on the FCC phase is rich in Cr_2_O_3_, which is usually dense and provides better protection. Therefore, corrosion is more likely to initiate in the BCC phase. The micro-galvanic coupling effect between the FCC and BCC phases accelerates the preferential dissolution of the BCC phase, which acts as the anode, resulting in the formation of stable pits under the locally attacked passive film (Figure 11b).

## 4. Conclusions

In this paper, we investigated the influence of post-heat treatment on the microstructure and corrosion property of AlCoCrFeNi_2.1_ EHEA prepared by LMD, and the following conclusions can be obtained:

1. The AlCoCrFeNi_2.1_ EHEA, whether heat-treated or not, exhibited typical dual-phase lamellar structure compromising FCC and BCC phases. With the increase in temperature of the heat treatment, the volume fraction of the FCC phase and interlamellar spacing both increased. The phase constitution obtained after heat treatment at 800 °C was the closest to the equilibrium state of as-cast AlCoCrFeNi_2.1_ EHEA.
2. As the heating temperature was elevated, the resistance of the AlCoCrFeNi_2.1_ EHEA to uniform corrosion was gradually enhanced. This was ascribed to the consistent increase in the volume fraction of the FCC phase in the alloy with the increasing heating temperature. The sample heat-treated at 1000 °C exhibited best resistance to uniform corrosion.
3. Potentiodynamic polarization and immersion tests both suggest that the samples heat-treated at 800 and 1000 °C suffered more severe pitting corrosion than the as-prepared and 600 °C-treated samples. It was found that heating at temperatures above 800 °C resulted in more pronounced enrichment of Al in the BCC phase, which rendered the Al_2_O_3_-rich passive film more susceptible to Cl^−^ ions attack.

## Figures and Tables

**Table 1:** Composition of AlCoCrFeNi_2.1_ alloy powders, at.%.

Element	Al	Co	Cr	Fe	Ni
Nominal	16.4	16.4	16.4	16.4	34.4
Actual	16.8 ± 0.3	16.2 ± 0.3	16.4 ± 0.3	16.2 ± 0.3	34.4 ± 0.3

**Table 2:** Heat treatment conditions of different AlCoCrFeNi_2.1_ samples.

Sample #	Heating Temperature/°C	Holding Time/h
1	-	-
2	600	4
3	800	4
4	1000	4

**Table 3:** EDS results of the four samples, at.%.

Phase	Sample		Al	Co	Cr	Fe	Ni
BCC		1#	31.4 ±0.4	12.6 ± 0.3	9.7 ± 0.3	9.0 ± 0.2	37.3 ± 0.3
		2#	30.0 ± 0.3	12.7 ± 0.4	9.9 ± 0.2	9.7 ± 0.3	37.7 ± 0.2
		3#	32.1 ± 0.3	10.7 ± 0.2	9.3 ± 0.4	8.6 ± 0.4	39.3 ± 0.2
		4#	36.4 ± 0.3	9.9 ± 0.3	5.7 ± 0.3	7.3 ± 0.3	40.7 ± 0.3
FCC		1#	13.9 ± 0.3	18.0 ± 0.4	19.8 ± 0.3	16.5 ± 0.3	31.8 ± 0.3
		2#	13.7 ± 0.4	17.6 ± 0.2	20.2 ± 0.3	15.8 ± 0.4	32.7 ± 0.4
		3#	12.4 ± 0.3	17.6 ± 0.4	20.1 ± 0.4	16.1 ± 0.2	33.8 ± 0.3
		4#	10.3 ± 0.3	19.9 ± 0.3	21.9 ± 0.3	17.3 ± 0.3	30.6 ± 0.2

**Table 4:** Electrochemical parameters of potentiodynamic polarization curves of the four samples and 304 stainless steel in 3.5 wt.% NaCl solution.

Sample	*E*_corr_/mV	*i*_corr_/(A·cm^−2^)	*E*_pit_/mV
1#	−369	2.5 × 10^−6^	189
2#	−375	1.7 × 10^−6^	194
3#	−346	1.4 × 10^−6^	47
4#	−297	3.0 × 10^−7^	99
304	−274	4.7 × 10^−7^	328

**Table 5:** The fitting results of EIS data of the four samples in 3.5 wt.% NaCl solution.

Sample	*R*_s_ (Ω·cm^2^)	*R*_f_ (Ω·cm^2^)	*R*_ct_ (Ω·cm^2^)	*Z*_CPE1_ (Ω^−1^·cm^−2^·s^n^)	*n*_1_	*Z*_CPE2_ (Ω^−1^·cm^−2^·s^n^)	*n*_2_
1#	23.41	30,588	1.28 × 10^5^	2.17 × 10^−5^	0.9393	2.64 × 10^−5^	0.8733
2#	24.78	32,290	3.04 × 10^5^	2.04 × 10^−5^	0.9585	2.81 × 10^−6^	0.5206
3#	25.16	37,475	1.73 × 10^5^	1.27 × 10^−5^	0.9186	8.92 × 10^−6^	0.4761
4#	26.1	60,753	6.89 × 10^5^	7.07 × 10^−6^	0.9155	3.74 × 10^−6^	0.5485

## Data Availability

The original contributions presented in this study are included in the article. Further inquiries can be directed to the corresponding author.

## References

[1] Cantor B., Chang I.T.H., Knight P., Vincent A.J.B. (2004). Microstructural development in equiatomic multicomponent alloys. Mater. Sci. Eng. A.

[2] Yeh J.W., Chen S.K., Lin S.J., Gan J.Y., Chin T.S., Shun T.T., Tsau C.H., Chang S.Y. (2004). Nanostructured High-Entropy Alloys with Multiple Principal Elements: Novel Alloy Design Concepts and Outcomes. Adv. Eng. Mater..

[3] Chen H., Lang L., Shang X., Dash S.S., He Y., King G., Zou Y. (2024). Anisotropic co-deformation behavior of nanolamellar structures in additively manufactured eutectic high entropy alloys. Acta Mater..

[4] Duan X., Han T., Guan X., Wang Y., Su H., Ming K., Wang J., Zheng S. (2023). Cooperative effect of Cr and Al elements on passivation enhancement of eutectic high-entropy alloy AlCoCrFeNi2.1 with precipitates. J. Mater. Sci. Technol..

[5] Lu Y., Dong Y., Guo S., Jiang L., Kang H., Wang T., Wen B., Wang Z., Jie J., Cao Z. (2014). A Promising New Class of High-Temperature Alloys: Eutectic High-Entropy Alloys. Sci. Rep..

[6] Lin G., Cai Z., Dong Y., Wang C., Hu J., Zhang P., Gu L. (2024). High-temperature oxidation behavior of AlCoCrFeNi2.1 eutectic high-entropy alloy: Microstructure evolution and microhardness. Mater. Charact..

[7] Gao P., Hu H., Xu J., You Y., Qi T., Zhang L., Cai W., Cao L., Li J. (2025). Tailored microstructure and mechanical properties of AlCoCrFeNi2.1 eutectic high-entropy alloy fabricated by laser-directed energy deposition with different energy densities. J. Mater. Res. Technol..

[8] Long X., Li Z., Yan J., Zhang T. (2023). Enhanced strength-ductility synergy of an AlCoCrFeNi2.1 eutectic high entropy alloy by ultrasonic vibration. J. Mater. Res. Technol..

[9] Gou S., Gao M., Shi Y., Li S., Fang Y., Chen X., Chen H., Yin W., Liu J., Lei Z. (2023). Additive manufacturing of ductile refractory high-entropy alloys via phase engineering. Acta Mater..

[10] Amar A., Wang M., Zhang L., Li J., Huang L., Yan H., Zhang Y., Lu Y. (2023). Additive manufacturing of VCoNi medium-entropy alloy: Microstructure evolution and mechanical properties. Addit. Manuf..

[11] Ostovari Moghaddam A., Shaburova N.A., Samodurova M.N., Abdollahzadeh A., Trofimov E.A. (2021). Additive manufacturing of high entropy alloys: A practical review. J. Mater. Sci. Technol..

[12] Liu Z., Zhao D., Wang P., Yan M., Yang C., Chen Z., Lu J., Lu Z. (2022). Additive manufacturing of metals: Microstructure evolution and multistage control. J. Mater. Sci. Technol..

[13] Hemmasian Ettefagh A., Guo S., Raush J. (2021). Corrosion performance of additively manufactured stainless steel parts: A review. Addit. Manuf..

[14] Wang S., Li Y., Zhang D., Yang Y., Marwana Manladan S., Luo Z. (2022). Microstructure and mechanical properties of high strength AlCoCrFeNi2.1 eutectic high entropy alloy prepared by selective laser melting (SLM). Mater. Lett..

[15] Vikram R.J., Murty B.S., Fabijanic D., Suwas S. (2020). Insights into micro-mechanical response and texture of the additively manufactured eutectic high entropy alloy AlCoCrFeNi_2.1_. J. Alloys Compd..

[16] Huang L., Sun Y., Chen N., Luan H., Le G., Liu X., Ji Y., Lu Y., Liaw P.K., Yang X. (2022). Simultaneously enhanced strength-ductility of AlCoCrFeNi2.1 eutectic high-entropy alloy via additive manufacturing. Mater. Sci. Eng. A.

[17] Zhou L., Zhang J., Feng G., Chen Y., Xie Y., Zhang J., Li G., Zhou T., Zhou Y., Duan F. (2025). Homogenization of AlCoCrFeNi_2.1_ eutectic high entropy with improved corrosion resistance fabricated by selective laser melting. Corros. Sci..

[18] Luo W., Yuan X., Zhang Z., Cheng C., Liu H., Qiu H., Cheng X. (2025). Effect of volumetric energy density on the mechanical properties and corrosion resistance of laser-additive-manufactured AlCoCrFeNi2.1 high-entropy alloys. J. Alloys Compd..

[19] Du L., Ding H., Xie Y., Ji L., Chen W., Xu Y. (2025). Effect of Laser Energy Density on Microstructures and Properties of Additively Manufactured AlCoCrFeNi_2.1_ Eutectic High-Entropy Alloy. Acta Metall. Sin. (Engl. Lett.).

[20] Lan L., Wang W., Cui Z., Sing S.L. (2024). Mechanical, materials, and physicochemical effects on the high-temperature tribological behaviour of laser additive manufacturing AlCoCrFeNi2.1 eutectic high-entropy alloys. Virtual Phys. Prototyp..

[21] Bijnavandi M.S., Dehghani K. (2025). Investigating the effect of hot -rolling and cold -rolling on the microstructure and mechanical properties of high entropy alloy AlCoCrFeNi2.1. J. Mater. Res. Technol..

[22] Jia Q., Gu D. (2014). Selective laser melting additive manufacturing of Inconel 718 superalloy parts: Densification, microstructure and properties. J. Alloys Compd..

[23] Wang H., He Q., Gao X., Shang Y., Zhu W., Zhao W., Chen Z., Gong H., Yang Y. (2024). Multifunctional High Entropy Alloys Enabled by Severe Lattice Distortion. Adv. Mater..

[24] Sui Q., Wang Z., Wang J., Yuan Q., Mao S., Yuan B., Xu S., Wen H., Xiao T., Wu Y. (2024). Strength-ductility balance of AlCoCrFeNi2.1 eutectic high-entropy alloy via additive manufacturing. J. Mater. Res. Technol..

[25] Yu T., Zhou G., Cheng Y., Hu F., Jiang T., Sun T., Shen Y., Zhou Y., Li J. (2023). Microstructure and properties of AlCoCrFeNi2.1 eutectic high entropy alloy manufactured by selective laser melting. Opt. Laser Technol..

[26] Wani I.S., Bhattacharjee T., Sheikh S., Bhattacharjee P.P., Guo S., Tsuji N. (2016). Tailoring nanostructures and mechanical properties of AlCoCrFeNi2.1 eutectic high entropy alloy using thermo-mechanical processing. Mater. Sci. Eng. A.

[27] Tang Z., Gao M.C., Diao H., Yang T., Liu J., Zuo T., Zhang Y., Lu Z., Cheng Y., Zhang Y. (2013). Aluminum Alloying Effects on Lattice Types, Microstructures, and Mechanical Behavior of High-Entropy Alloys Systems. JOM.

[28] Song L., Hu W., Liao B., Wan S., Kan L., Guo X. (2023). Corrosion behavior of AlCoCrFeNi_2.1_ eutectic high-entropy alloy in Cl^−^-containing solution. J. Alloys Compd..

[29] Li T., Swanson O.J., Frankel G.S., Gerard A.Y., Lu P., Saal J.E., Scully J.R. (2019). Localized corrosion behavior of a single-phase non-equimolar high entropy alloy. Electrochim. Acta.

[30] Duan J., Luo S., Hou C., Dong Y., Mao L. (2025). Effects of Cr replacing Fe on microstructure and properties of AlCoCrFeNi_2.1_ eutectic high entropy alloy. J. Mater. Res. Technol..

[31] Wang R., Zhang K., Davies C., Wu X. (2017). Evolution of microstructure, mechanical and corrosion properties of AlCoCrFeNi high-entropy alloy prepared by direct laser fabrication. J. Alloys Compd..

[32] Hasannaeimi V., Mukherjee S. (2019). Galvanic corrosion in a eutectic high entropy alloy. J. Electroanal. Chem..

[33] Tian W., Du N., Li S., Chen S., Wu Q. (2014). Metastable pitting corrosion of 304 stainless steel in 3.5% NaCl solution. Corros. Sci..

[34] Fu Y., Dai C., Luo H., Li D., Du C., Li X. (2021). The corrosion behavior and film properties of Al-containing high-entropy alloys in acidic solutions. Appl. Surf. Sci..

[35] Della Rovere C.A., Alano J.H., Silva R., Nascente P.A.P., Otubo J., Kuri S.E. (2012). Characterization of passive films on shape memory stainless steels. Corros. Sci..

[36] Erfani Mobarakeh S.A., Dehghani K. (2024). Microstructural evolutions and corrosion behavior of nanocomposite AlCoCrFeNi2.1 high-entropy alloy produced via friction stir processing. J. Mater. Res. Technol..

